# Synthesis, Structural Characterization, and Hirschfeld Surface Analysis of a New Cu(II) Complex and Its Role in Photocatalytic CO_2_ Reduction

**DOI:** 10.3390/molecules29091957

**Published:** 2024-04-24

**Authors:** Li-Hua Wang, Mohammad Azam, Xi-Hai Yan, Xi-Shi Tai

**Affiliations:** 1 College of Biology and Oceanography, Weifang University, Weifang 261061, China; 2 Department of Chemistry, College of Science, King Saud University, P.O. Box 2455, Riyadh 11451, Saudi Arabia; 3 College of Chemistry and Chemical Engineering, Weifang University, Weifang 261061, China

**Keywords:** 6-phenylpyridine-2-carboxylic acid, 4-[5-(pyridin-4-yl)-1,3,4-oxadiazol-2-yl]pyridine, Cu(II) complex, synthesis, crystal structure, Hirschfeld surface analysis, photocatalytic CO_2_ reduction

## Abstract

A new Cu(II) complex, [CuL_1_L_2_(CH_3_COO)_2_(H_2_O)]·H_2_O, was synthesized by the reaction of Cu(CH_3_COO)_2_·H_2_O, 6-phenylpyridine-2-carboxylic acid (HL_1_), and 4-[5-(pyridin-4-yl)-1,3,4-oxadiazol-2-yl]pyridine (L_2_) in ethanol-water (*v*:*v* = 1:1) solution. The Cu(II) complex was characterized using elemental analysis, IR, UV-vis, TG–DTA, and single-crystal X-ray analysis. The fluorescence properties of the copper complex were also evaluated. The structural analysis results show that the Cu(II) complex crystallizes in the triclinic system with space group *P*-1. The Cu(II) ion in the complex is five-coordinated with one O atom (O2) and one N atom (N1) from one 6-phenylpyridine-2-carboxylate ligand (L_1_), one N atom (N2) from 4-[5-(pyridin-4-yl)-1,3,4-oxadiazol-2-yl]pyridine ligand (L_2_), one O atom (O4) from acetate, and one O atom (O5) from a coordinated water molecule, and it adopts a distorted trigonal bipyramidal geometry. Cu(II) complex molecules form a two-dimensional layer structure through intramolecular and intermolecular O-H^…^O hydrogen bonding. The two-dimensional layer structures further form a three-dimensional network structure by π-π stacking interactions of aromatic rings. The analysis of the Hirschfeld surface of the Cu(II) complex shows that the H^…^H contacts made the most significant contribution (46.6%) to the Hirschfeld surface, followed by O^…^H/H^…^O, N^…^H/H^…^N and C^…^H/H^…^C contacts with contributions of 14.2%, 13.8%, and 10.2%, respectively. In addition, the photocatalytic CO_2_ reduction using Cu(II) complex as a catalyst is investigated under UV-vis light irradiation. The findings reveal that the main product is CO, with a yield of 10.34 μmol/g and a selectivity of 89.4% after three hours.

## 1. Introduction

**Figure 1:**
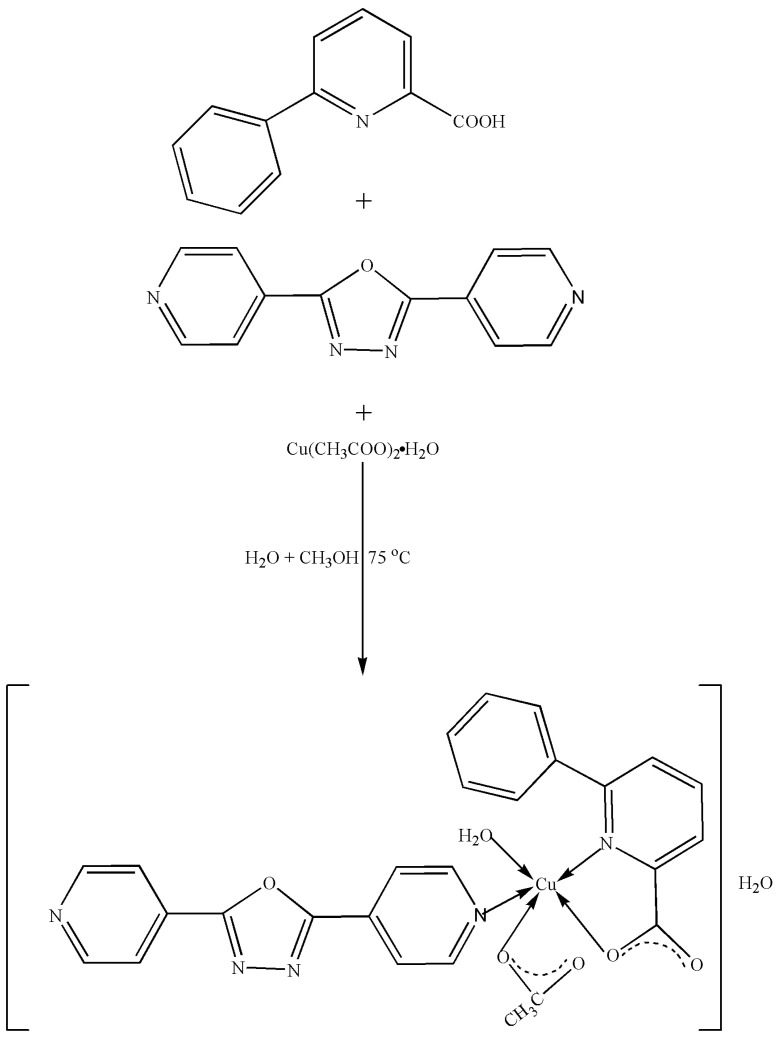
Synthesis of Cu(II) complex.

With the rapid development of the fossil fuel industry, large amounts of CO_2_ have been excessively emitted, causing climate changes, such as droughts, typhoons, acid rains, cold waves, high-temperature heat waves, and dust storms [1]. Therefore, there is an urgent need to find highly efficient CO_2_ conversion technologies. Reducing CO_2_ by photocatalytic processes is a promising strategy for achieving sustainability, which not only diminishes CO_2_ emissions but also produces valuable chemicals and fuels, making a substantial contribution to environmental remediation to address the urgent concerns regarding climate change and the ultimate exhaustion of fossil fuel reserves [2,3]. In addition, several technologies that transform CO_2_ into high-value-added products, such as CO, CH_4_, CH_3_OH, ethylene, ethanol, and HCOOH, have been investigated. Various metal oxide composite catalysts are widely used in photocatalytic CO_2_ reduction due to their unique properties, high stability, and low cost [4,5,6,7,8]. Cu catalysts for the synthesis of multi-carbon products exhibit a series of consistent motifs, such as oxidation, a high surface area, nanostructuring, and specific faceting, which promote the production of ethylene and ethanol at consistently lower over-potentials, with Faradaic efficiencies approaching 50–60%. However, these catalysts have a short lifespan and limitations in terms of the current density of the substrate [9]. Metal-semiconductor catalysts have been proven to widen the light absorption range of light-driven catalyst materials, and the presence of metal particles not only enhances the optical absorption capability but also provides active sites for the activation of CO_2_; however, the cost of these catalysts is very high because many of them contain precious metals [10]. Despite their potential, these systems have not been widely used because of several limitations that greatly limit their practical applications, including their inefficiency in converting energy, high charge recombination, and limited capacity to absorb visible light [11]. Since the initial report on photocatalytic CO_2_ reduction using fac-[ReICl(bpy)(CO)_3_] [12], researchers have constructed numerous effective systems for reducing CO_2_ using noble metal complexes by finetuning the features of the catalytic systems, and they have developed molecular catalysts, such as dinuclear rhenium-bipyridine assemblies, trinuclear ruthenium polyazine-GOphen compounds and ruthenium trisphenanthroline assemblies, as photocatalysts for the conversion of CO_2_ [13,14,15]. However, due to the high cost of these noble metal complexes, the use of earth-abundant metal complexes as catalysts for photocatalytic CO_2_ reduction has attracted increasing attention from chemists and material scientists. Among these complexes, Co(II), Mn(II), Fe(II), Cu(I), and rare earth ions containing complexes have been used for photocatalytic CO_2_ reduction [16,17,18,19,20,21,22,23,24]. However, to date, there are very few reports on the photocatalytic CO_2_ reduction by Cu(II) complexes [25,26,27]. 6-Phenylpyridine-2-carboxylic acid is an excellent ligand and can form structurally stable, coordination-diverse complexes with many metal ions [28,29]. To further enhance the application of metal complexes in the photocatalytic reduction of CO_2_, and to explore the synergistic action of metal ions and ligands in metal complexes with photocatalytic CO_2_ reduction (rather than the role of simple metal ions or the role of simple ligands), we have synthesized a new Cu(II) complex by one-pot method using Cu(CH_3_COO)_2_·H_2_O, 6-phenylpyridine-2-carboxylic acid (HL_1_), and 4-[5-(pyridin-4-yl)-1,3,4-oxadiazol-2-yl]pyridine (L_2_) in an ethanol-water solution. The isolated Cu(II) complex was thoroughly characterized using elemental analysis, IR, UV-vis, TG–DTA, and single-crystal X-ray analysis. In addition, the fluorescence behavior of the complex was also investigated. Moreover, the Cu(II) ion in the complex is five-coordinated with one O atom and one N atom from one 6-phenylpyridine-2-carboxylate ligand (L_1_), one N atom from 4-[5-(pyridin-4-yl)-1,3,4-oxadiazol-2-yl]pyridine ligand (L_2_), one O atom from acetate, and one O atom from coordinated water molecule, and it adopts a distorted trigonal bipyramid geometry. Furthermore, the photocatalytic reduction of CO_2_ by Cu(II) complex was investigated using UV-vis light irradiation, and the results indicated that the main product formed is CO, with a yield of 10.34 μmol/g and a selectivity of 89.4% after three hours. CO can be widely used in the metallurgical industry as a reducing agent, gas fuel, etc. The synthesis of the Cu(II) complex is shown in Figure 1.

## 2. Results and Discussion

### 2.1. Infrared Spectra

**Figure 2:**
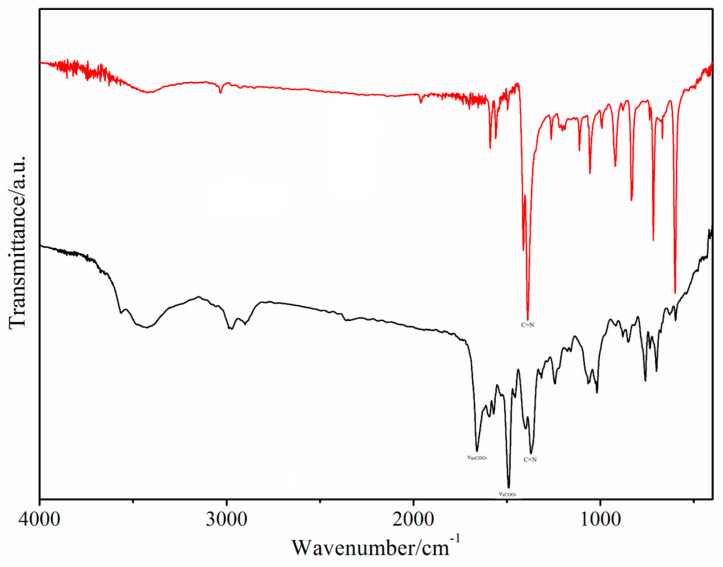
The infrared spectra of the Cu(II) complex (black) and 4-[5-(pyridin-4-yl)-1,3,4-oxadiazol-2-yl]pyridine (L_2_) ligand (red).

The infrared spectra of the Cu(II) complex and 4-[5-(pyridin-4-yl)-1,3,4-oxadiazol-2-yl]pyridine (L_2_) ligand are shown in Figure 2. The free 6-phenylpyridine-2-carboxylic acid (HL_1_) ligand shows characteristic bands at 1646 (ν_as_COO^−^) and 1575 (ν_s_COO^−^) cm^−1^ [30], while the free 4-[5-(pyridin-4-yl)-1,3,4-oxadiazol-2-yl]pyridine (L_2_) ligand shows an important band at 1388 cm^−1^ (νC=N). In Cu(II) complex, these bands are observed at ca. 1660, 1489, and 1371 cm^−1^, respectively, indicating the coordination of both the L_1_ ligand and L_2_ ligands with the Cu(II) ion. The difference between νasCOO^−^ and νsCOO^−^|ν_as_COO^−^ − ν_s_COO^−^| = 171 cm^−1^ suggests that the COO^−^ group of 6-phenylpyridine-2-carboxylic acid (HL_1_) ligand in the Cu(II) complex adopts a monodentate coordinated mode. In addition, a broad absorption band at ca. 3481 cm^−1^ is assigned to the ν(OH), indicating the presence of water molecules in the Cu(II) complex. The IR results are consistent with the X-ray single-crystal diffraction data of the Cu(II) complex.

### 2.2. UV-Vis Spectrum

**Figure 3:**
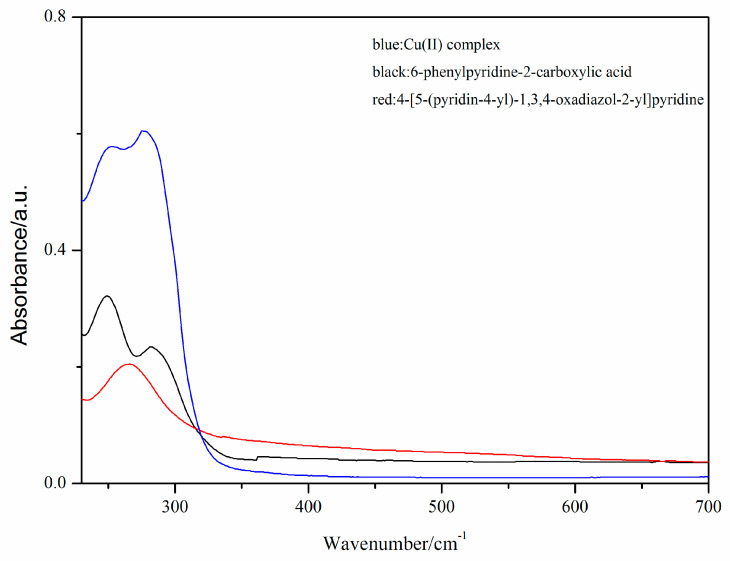
The UV-vis absorption spectra of free ligands and Cu(II) complex.

The UV-vis spectra of the free 6-phenylpyridine-2-carboxylic acid (HL_1_) ligand, the 4-[5-(pyridin-4-yl)-1,3,4-oxadiazol-2-yl]pyridine (L_2_) ligand and the Cu(II) complex are shown in Figure 3. The free 6-phenylpyridine-2-carboxylic acid (HL_1_) ligand exhibits two absorption bands at 249 and 282 cm^−1^, while the free 4-[5-(pyridin-4-yl)-1,3,4-oxadiazol-2-yl]pyridine (L_2_) ligand exhibits one absorption band at 266 cm^−1^. The Cu(II) complex shows bands at ca. 253 and 276 cm^−1^, which may be attributed to the π-π* transitions of L_1_ and L_2_ ligands. The variations in the UV absorption peaks between the Cu(II) complex and L_1_ and L_2_ ligands indicate the coordination of L_1_ and L_2_ ligands to the Cu(II) ion, which agrees well with the results of the infrared spectra.

### 2.3. Thermogravimetric Analysis

**Figure 4:**
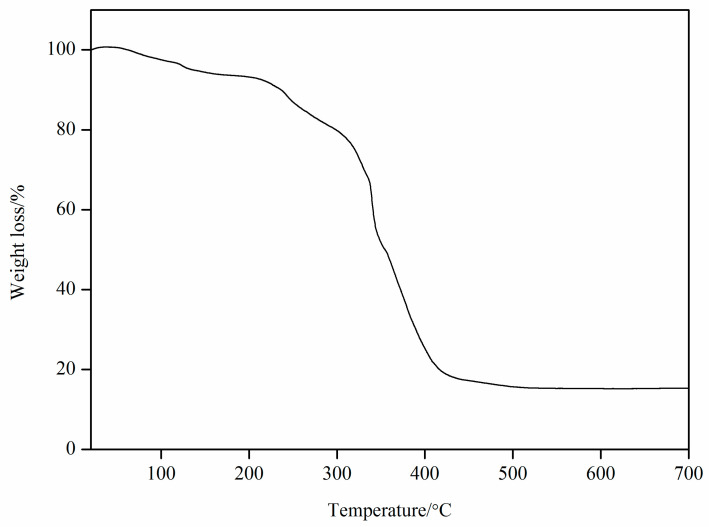
Thermal stability curve of Cu(II) complex.

The thermogravimetric analysis of the Cu(II) complex was performed in the air atmosphere with a heating rate of 5 °C/min (room temperature to 700 °C). The thermal stability curve of the Cu(II) complex is shown in Figure 4, and it shows that its decomposition went through two distinct phases: The first stage occurs at 25–200 °C, corresponding to a weight loss of 6.20%, which is likely attributed to the loss of two water molecules (6.19%). The second stage occurs at 200–700 °C, corresponding to a weight loss of 15.30%, which may be due to the continuous decomposition of 6-phenylpyridine-2-carboxylate (L_1_) ligand, 4-[5-(pyridin-4-yl)-1,3,4-oxadiazol-2-yl]pyridine (L_2_) ligand, and acetate. The final residue identified was CuO (Found: 15.30%, Calculated: 13.77%). 

### 2.4. Structural Description of Cu(II) Complex

**Figure 5:**
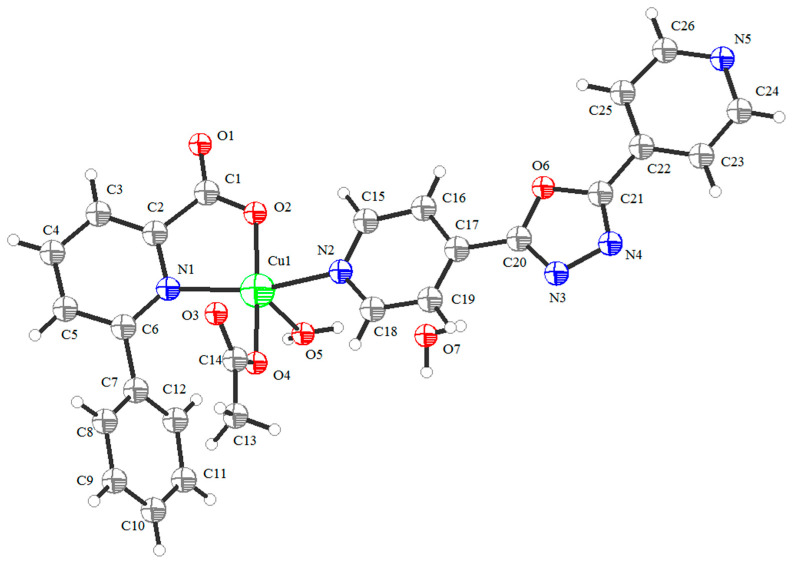
The molecular structure of Cu(II) complex. Red: O atoms, blue: N atoms, green: Cu atom, gray: C atoms.

**Figure 6:**
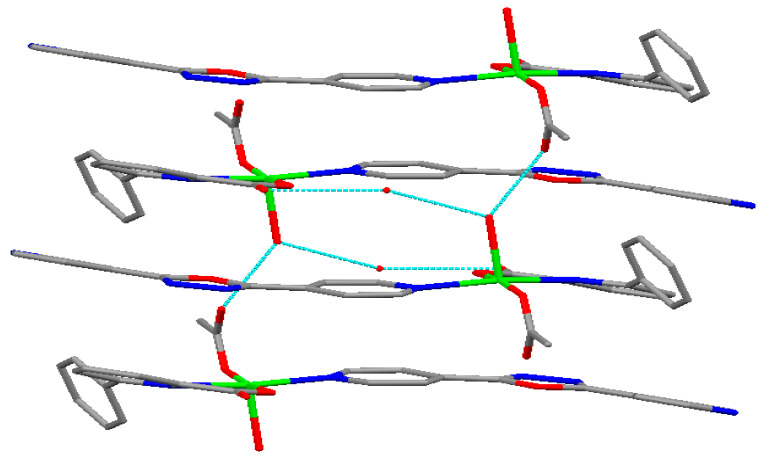
The two-dimensional layered structure of Cu(II) complex formed by hydrogen bonds. Red: O atoms, blue: N atoms, green: Cu atom, gray: C atoms.

**Figure 7:**
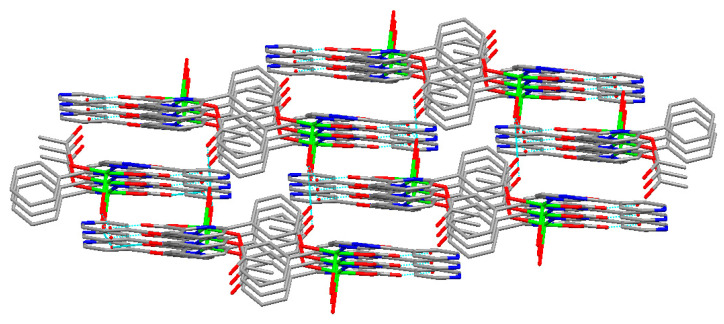
The three-dimensional network structure of Cu(II) complex stacked by two-dimensional layered structure. Red: O atoms, blue: N atoms, green: Cu atom, gray: C atoms.

The Cu(II) complex crystallizes in a triclinic system with space group *P*-1. Its structure comprises one Cu(II) ion, one 4-[5-(pyridin-4-yl)-1,3,4-oxadiazol-2-yl]pyridine (L_2_) ligand, one 6-phenylpyridine-2-carboxylate (L_1_) ligand, one acetate, one coordinated water molecule, and one uncoordinated water molecule. The molecular structure of the Cu(II) complex is shown in Figure 5. The selected bond lengths (Å) and angles (°) for the Cu(II) complex are listed in Table 1. The two-dimensional layered structure of the Cu(II) complex formed by hydrogen bonds is shown in Figure 6. Additionally, a three-dimensional network structure of Cu(II) complex stacked by a two-dimensional layered structure is shown in Figure 7. As shown in Figure 5, the Cu(II) ion exhibits a five-coordinated geometry, surrounded by one N atom (N1) and one carboxylate O atom (O2) from the same 6-phenylpyridine-2-carboxylate (L_1_) ligand, one N atom (N2) from 4-[5-(pyridin-4-yl)-1,3,4-oxadiazol-2-yl]pyridine (L_2_) ligand, one O atom (O4) from one acetate, and one O atom (O5) from one coordinated water molecule, and they form a trigonal bipyramidal geometry with an O4-Cu1-O2 bond angle of 172.04(7)°. The bond angles around the Cu(II) ion are N1-Cu1-N2 (165.99(7)°), N1-Cu1-O5 (103.25(6)°), N2-Cu1-O5 (86.56(7)°), with a total sum of 355.8°, showing that the N1, N2 and O5 atoms are at the equatorial plane. The dihedral angle of ring 1 (N1-C2-C3-C4-C5-C6-N1) and ring 2 (C7-C8-C9-C10-C11-C12-C7) is 49.97°, indicating that the 6-phenylpyridine-2-carboxylate (L_1_) ligand is not coplanar, and that of ring 3 (N2-C15-C16-C17-C19-C18-N2) and ring 4 (O6-C20-N3-N4-C21-O6) is 5.05° while that of ring 5 (N5-C24-C23-C22-C25-C26-N5) and ring 4 (O6-C20-N3-N4-C21-O6) is 9.49°, indicating that the 4-[5-(pyridin-4-yl)-1,3,4-oxadiazol-2-yl]pyridine (L_2_) ligand is almost coplanar. The results of the dihedral angles show that the Cu(II) complex molecule is not coplanar. The bond distances of Cu1-N1, Cu1-N2, Cu1-O2, Cu1-O4, and Cu1-O5 are 2.1179(17), 2.0832(18), 1.9216(16), 1.9203(14), and 2.2628(16) Å, respectively, consistent with literature reports [29,31]. In the Cu(II) complex, both the 6-phenylpyridine-2-carboxylate (L1) ligand and acetate adopt a monodentate coordinated mode, which is in agreement with the infrared spectra results. The Cu(II) complex molecules form a two-dimensional layered structure by O-H^…^O through hydrogen bonds including uncoordinated water molecules, coordinated water molecules, carboxylate O atoms of 6-phenylpyridine-2-carboxylate (L_1_) ligand, and O atoms of acetate (Figure 6). These two-dimensional layer structures further form a three-dimensional network structure by π-π stacking interactions of aromatic rings (Figure 7). The hydrogen bonds of the Cu(II) complex are listed in Table 2.

### 2.5. Hirschfeld Surface Analysis of Cu(II) Complex

**Figure 8:**
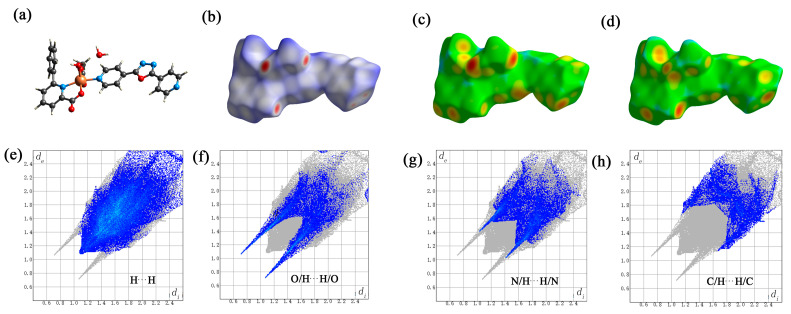
The Hirschfeld surface of the Cu(II) complex. the original crystal structure unit (**a**) *d*_norm_ (**b**), *d*_i_ (**c**), *d*_e_ (**d**); and the top three interactions (H^…^H (**e**), O/H^…^H/O (**f**), N/H^…^H/N (**g**) and C/H^…^H/C (**h**)).

The Hirschfeld surface of the Cu(II) complex was analyzed using the Crystal Explorer software 21.5. Figure 8 displays the original crystal structure unit (a), the Hirschfeld surfaces mapped over *d*_norm_, *d*_i_ and *d*_e_ of the crystal (b–d), and the two-dimensional (2D) fingerprint plots representing the overall and top four interactions (H^…^H, O^…^H/H^…^O, N^…^H/H^…^N and C^…^H/H^…^C) (e–h). Based on the calculations, it can be concluded that the H^…^H contacts represented the largest contribution (46.6%) to the Hirschfeld surface, followed by O^…^H/H^…^O, N^…^H/H^…^N and C^…^H/H^…^C contacts with contributions of 14.2%, 13.8% and 10.2%, respectively. It is worth noting that the π-π stacking interactions play a minor role in the formation, representing 6.6% of the Hirschfeld surface contribution for the C^…^C contacts.

### 2.6. Fluorescence Studies

**Figure 9:**
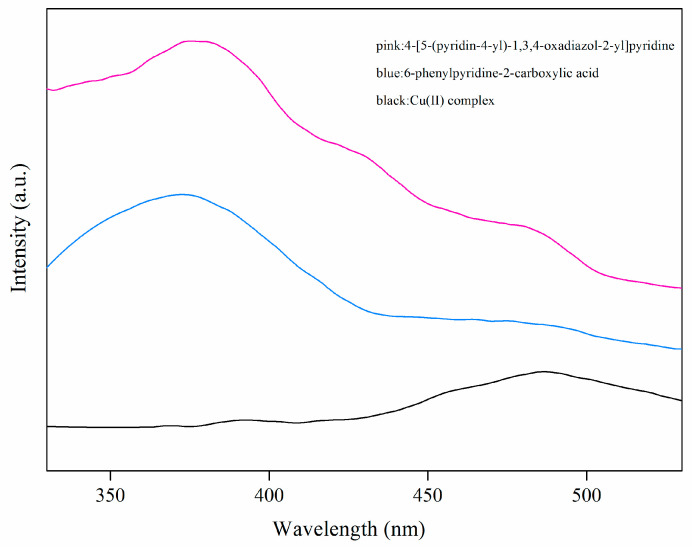
The emission spectra of the Cu(II) complex and the ligands 4-[5-(pyridin-4-yl)-1,3,4-oxadiazol-2-yl]pyridine and 6-phenylpyridine-2-carboxylic acid in ethanol.

The fluorescence behavior of the Cu(II) complex, along with the ligands 4-[5-(pyridin-4-yl)-1,3,4-oxadiazol-2-yl]pyridine and 6-phenylpyridine-2-carboxylic acid, were explored in ethanol. The excitation and emission slit widths were 2.5 nm. The emission spectra for each compound are illustrated in Figure 9. In the case of the Cu(II) complex, a weak luminescent emission peak was observed at 486 nm upon excitation at 296 nm. Meanwhile, the ligands 4-[5-(pyridin-4-yl)-1,3,4-oxadiazol-2-yl]pyridine and 6-phenylpyridine-2-carboxylic acid exhibited luminescent emission peaks at 384 nm and 365 nm, respectively, under the same excitation conditions. It is worth mentioning that the maximum emission peak for each compound was observed to be red-shifted in comparison to the free ligands.

### 2.7. Photocatalytic CO_2_ Reduction Activity of Cu(II) Complex

**Figure 10:**
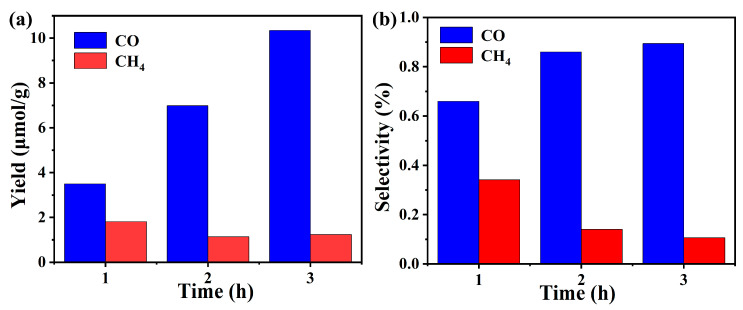
(**a**) Photocatalytic CO_2_ reduction performance and (**b**) product selectivity of Cu(II) complex catalyst.

We are concerned here with exploring the potential application of the Cu(II) complex as a catalyst in the photocatalytic reduction of CO_2_. The experimental findings shown in Figure 10 clearly exhibit the performance of the Cu(II) complex as a catalyst ((a) photocatalytic CO_2_ reduction performance and (b) product selectivity of Cu(II) complex catalyst). The main product is CO, and as the reaction period extended, the yield gradually increased. Specifically, after three hours of UV-Vis light irradiation, the yield reached 10.34 μmol (CO)/g (catalyst). Moreover, methane products have been detected in trace amounts, with an approximate output of 1.22 μmol (CH_4_)/g (catalyst) after three hours of UV-vis light irradiation. The CO selectivity is high, at 89.4%. To prove whether the Cu(II) complex catalyst changed before and after the catalysis, we also tested the elemental analysis of the Cu(II) complex catalyst after the catalytic reaction, and the results showed that the catalyst did not change (Found: C, 53.49%, H, 4.12%, N, 11.71%). Our investigation involved the use of two metal complexes as catalysts for the photocatalytic CO_2_ reduction: one is a dinuclear Gd(III) complex constructed by 6-phenylpyridine-2-carboxylic acid (L) and 1,10-phenanthroline ligands, [Gd_2_(L)_4_(Phen)_2_(H_2_O)_2_(DMF)_2_]·2H_2_O·2Cl (1) [24], and the other is a Gd(III) coordination polymer constructed using 6-phenylpyridine-2-carboxylic acid (L_1_) and 4,4′-bipyridine ligands, {[Gd(L_1_)_3_(H_2_O)_2_]∙L_2_}_n_ (2) [29]. The results indicate that the primary catalytic products using complex (1) and complex (2) as catalysts were all CO, with yields and a selectivity of 22.1 μmol/g and 78.5% and of 60.3 μmol/g and 100%, respectively. A comparison of the catalytic activity among the three complexes reveals that the yield follows the following order: complex (2) > complex (1) > Cu(II) complex (this work); meanwhile, the selectivity follows the following order: complex (2) > Cu(II) complex (this work) > complex (1). This suggests that both the central metal ion and the ligand play crucial roles in influencing the catalytic activity of the complex.

## 3. Experimental Section

### 3.1. Materials and Measurements

Cu(CH_3_COO)_2_·H_2_O, 6-phenylpyridine-2-carboxylic acid (HL_1_), 4-[5-(pyridin-4-yl)-1,3,4-oxadiazol-2-yl]pyridine (L_2_), and NaOH were purchased commercially from Jilin Chinese Academy of Sciences-Yanshen Technology Co., Ltd. (Jilin, China) and used as received without further purification. C, H and N analyses were performed using a Vario III EL elemental analyzer (Elementar, Hanau, Germany). IR spectra were recorded on a Tianjin Gangdong FTIR-850 spectrophotometer (Tianjin, China) (KBr discs, 4000–400 cm^–1^). UV-vis spectra in the 190–700 nm region were carried out with a PERSEE T9 spectrophotometer (Beijing, China) in water with quartz cuvettes of 1 cm path length. TG–DTA was recorded by a HENVEN HCT-2 thermal analyzer (Beijing, China). The Hirschfeld surface of the Cu(II) complex was calculated by the Crystal Explorer software [32]. Fluorescence measurements were made on a PE LS-55 fluorescence spectrophotometer equipped with quartz cuvettes of 1 cm path length (PerkinElmer, Waltham, MA, USA). The excitation and emission slit widths were 2.5 nm. X-ray diffractions of the Cu(II) complex were collected on a Bruker CCD area detector (SuperNova, Billerica, MA, USA, Dual, Cu at zero, 296.15 K, multi-scan). Samples of the compounds are available from the authors.

### 3.2. Synthesis of Cu(II) Complex

A mixture of 6-phenylpyridine-2-carboxylic acid (HL_1_) (0.0996 g, 0.5 mmol), 4-[5-(pyridin-4-yl)-1,3,4-oxadiazol-2-yl]pyridine (L_2_) (0.1000 g, 0.5 mmol), NaOH (0.020 g, 0.5 mmol), and Cu(CH_3_COO)_2_·H_2_O (0.0998 g, 0.5 mmol) was added to the solution of 20 mL water-ethanol (*v*:*v* = 1:1) with stirring. The mixture was heated at 76 °C for 4 h and stirred continuously for 2 h at room temperature. The blue block crystals of the Cu(II) complex grew out from the filtrate after 10 days by evaporation. Elemental analysis calcd for [CuL_1_L_2_(CH_3_COO)_2_(H_2_O)]·H_2_O: C, 53.70%, H, 3.96%, N, 12.05%; Found: C, 53.46%, H, 4.19%, N, 11.76%.

### 3.3. Crystal Structure Determination

X-ray diffraction data for the single crystal (dimensions of 0.15 mm × 0.13 mm × 0.11 mm) of the Cu(II) complex were collected and mounted on a SuperNova, Dual, Cu at zero Bruker Smart CCD diffractometer. Data were collected at 296.10(10) K by using a graphite-monochromator with Mo*Kα* radiation. Data collection and absorption correction were carried out by the Olex2 [33]. The structure was solved by the SHELXS program [34] and refined by the Least-Squares minimization techniques SHELXL [35] program. The crystal structural parameters for the Cu(II) complex are listed in Table 3.

Crystallographic data for the structure reported in this paper have been deposited in the Cambridge Crystallographic Data Centre as supplementary publication No. CCDC 2333570. The CIF file can be obtained conveniently from the website: https://www.ccdc.cam.ac.uk/structures (accessed on 19 February 2024).

### 3.4. Photocatalytic CO_2_ Reduction Test

A total of 7 mg of Cu(II) complex catalyst and 50 mL deionized water were mixed into a quartz reactor. Then, the high-purity CO_2_ gas was passed into the suspension and maintained at a temperature of 20 °C. After 15 min, the reactor was sealed, and a xenon lamp was turned on (Beijing Trusttech Co., Ltd., Beijing, China). The gas analysis was conducted using a gas chromatograph equipped with a FID detector and Propark Q column (Shandong Huifen Instrument Co., Ltd., Zaozhuang, China).

## 4. Conclusions

A newly designed Cu(II) complex has been successfully synthesized and thoroughly characterized using various analytical techniques, including elemental analysis, infrared spectroscopy (IR), UV-visible spectroscopy (UV-vis), thermogravimetric–differential thermal analysis (TG–DTA), and single-crystal X-ray analysis. The fluorescence properties of the copper complex were also studied. Additionally, Hirschfeld surface analyses have been conducted on the Cu(II) complex. Furthermore, the photocatalytic activity of the Cu(II) complex in CO_2_ reduction under UV-visible light irradiation was investigated. The results revealed that CO is the primary product, with yields of 10.34 μmol/g and a selectivity of 89.4% after three hours. The findings presented here serve as a valuable reference for the potential design and synthesis of metal complex catalysts for photocatalytic CO_2_ reduction applications.

## Figures and Tables

**Table 1:** Selected bond lengths (Å) and bond angles (°) for Cu(II) complex.

Bond	*d*	Angle	(°)
Cu1-O2	1.9216 (16)	O2-Cu1-O5	96.77 (8)
Cu1-O4	1.9203 (14)	O2-Cu1-N1	81.18 (7)
Cu1-O5	2.2628 (16)	O2-Cu1-N2	87.83 (7)
Cu1-N1	2.1179 (17)	O4-Cu1-O2	172.04 (7)
Cu1-N2	2.0832 (18)	O4-Cu1-O5	91.10 (7)
		O4-Cu1-N1	98.22 (6)
		O4-Cu1-N2	91.47 (6)
		N1-Cu1-O5	103.25 (6)
		N2-Cu1-O5	86.56 (7)
		N2-Cu1-N1	165.99 (7)
		C1-O2-Cu1	118.87 (16)
		C2-N1-Cu1	107.96 (14)
		C6-N1-Cu1	132.89 (13)
		O1-C1-O2	124.7 (2)
		C20-O6-C21	102.67 (16)
		C15-N2-Cu1	120.08 (15)
		C18-N2-Cu1	122.95 (15)
		C20-N3-N4	105.93 (18)
		C21-N4-N3	106.40 (18)
		C14-O4-Cu1	115.06 (12)

**Table 2:** Hydrogen bonds in Cu(II) complex.

Donor-H	Acceptor	D-H (Å)	H^…^A (Å)	D^…^A (Å)	D-H^…^A (°)
O5-H5A	O3 ^#1^	0.84	1.92	2.7516 (1)	172
O5-H5B	O7 ^#1^	0.85	2.02	2.8221 (1)	159
O7-H7A	O1 ^#2^	0.85	2.18	2.9809 (1)	157

Symmetric operation code: #1: 1 + *x*, 1 − *y*, 1 − *z*; #2: 1 + *x*, *y*, *z*.

**Table 3:** The crystal structural parameters for Cu(II) complex.

Empirical Formula	C_26_H_23_CuN_5_O_7_
Formula weight	581.03
Temperature/*K*	296.10 (10)
Crystal system	triclinic
Space group	*P*-1
*a*/Å	7.11322 (11)
*b*/Å	11.62617 (17)
*c*/Å	16.2290 (3)
*α*/°	96.8735 (13)
*β*/°	98.3307 (13)
*γ*/°	92.0443 (12)
Volume/Å^3^	1316.50 (4)
*Z*	2
ρ_calc_, mg/mm^3^	1.466
μ/mm^−1^	1.643
*S*	1.058
*F* (000)	598
Index ranges	−8 ≤ *h* ≤ 7, −14 ≤ *k* ≤ 14, −20 ≤ *l* ≤ 20
Reflections collected	37,707
Independent reflections	5238 [R (int) = 0.0485]
Data/restraints/parameters	5238/4/363
Goodness-of-fit on *F*^2^	1.058
Refinement method	Full-matrix least-squares on *F*^2^
Final *R* indexes [*I* ≥ 2σ (*I*)]	R_1_ = 0.0410, *wR*_2_ = 0.1218
Final *R* indexes [all data]	R_1_ = 0.0463, *wR*_2_ = 0.1158

## Data Availability

Data are contained within the article.

## References

[1] Shang Z.A., Feng X.T., Chen G.Z., Qin R., Han Y.H. (2023). Recent advances on single-atom catalysts for photocatalytic CO_2_ reduction. Small.

[2] Protti S., Albini A., Serpone N. (2014). Photocatalytic generation of solar fuels from the reduction of H_2_O and CO_2_: A look at the patent literature. Phys. Chem. Chem. Phys..

[3] Centi G., Perathoner S. (2010). Towards solar fuels from water and CO_2_. ChemSusChem.

[4] Li M.D., Wang Z.M., Qi J., Yu R.B. (2023). Progress in the construction of metal oxide heterojunctions and their application in photocatalytic CO_2_ reduction. Chem. J. Chin. Univ..

[5] Gao X.Q., Cao L.L., Chang Y., Yuan Z.Y., Zhang S.X., Liu S.J., Zhang M.T., Fan H., Jiang Z.Y. (2023). Improving the CO_2_ Hydrogenation Activity of Photocatalysts via the Synergy between Surface Frustrated Lewis Pairs and the CuPt Alloy. ACS Sustain. Chem. Eng..

[6] Yin H.B., Li J.H. (2023). New insight into photocatalytic CO_2_ conversion with nearly 100% CO selectivity by CuO-Pd/HxMoO_3−y_ hybrids. Appl. Catal. B Environ..

[7] Heng Q.Q., Ma Y.B., Wang X., Wu Y.F., Li Y.Z., Chen W. (2023). Role of Ag, Pd cocatalysts on layered SrBi_2_Ta_2_O_9_ in enhancing the activity and selectivity of photocatalytic CO_2_ reaction. Appl. Surf. Sci..

[8] Shang X.F., Li G.J., Wang R.N., Xie T., Ding J., Zhong Q. (2023). Precision loading of Pd on Cu species for highly selective CO_2_ photoreduction to methanol. Chem. Eng. J..

[9] Ross M.B., De Luna P., Li Y., Dinh C.T., Kim D., Yang P., Sargent E.H. (2019). Designing materials for electrochemical carbon dioxide recycling. Nat. Catal..

[10] Yuan Z.M., Zhu X.L., Jiang Z.Y. (2023). Recent advances of constructing metal/semiconductor catalysts designing for photocatalytic CO_2_ hydrogenation. Molecules.

[11] Olowoyo J.O., Kumar M., Singhal N., Jain S.L., Babalola J.O., Vorontsov A.V., Kumar U. (2018). Engineering and modeling the effect of Mg doping in TiO_2_ for enhanced photocatalytic reduction of CO_2_ to fuels. Catal. Sci. Technol..

[12] Li N.X., Chen Y.M., Xu Q.Q., Yang Z. (2023). Photocatalytic reduction of CO_2_ to CO using manganese complexes with bipyridine modifed electron-donating groups. Catal. Lett..

[13] Fox A.R., Bart S.C., Meyer K., Cummins C.C. (2008). Towards uranium catalysts. Nature.

[14] Kumar P., Sain B., Jain S.L. (2014). Photocatalytic reduction of carbon dioxide to methanol using a ruthenium trinuclear polyazine complex immobilized on graphene oxide under visible light irradiation. J. Mater. Chem. A.

[15] Boston D.J., Xu C., Armstrong D.W., MacDonnell F.M. (2013). Photochemical reduction of carbon dioxide to methanol and formate in a homogeneous system with pyridinium catalysts. J. Am. Chem. Soc..

[16] Zhao K., Zhao S., Gao C., Qi J., Yin H., Wei D., Mideksa M.F., Wang X., Gao Y., Tang Z. (2018). Metallic cobalt–carbon composite as recyclable and robust magnetic photocatalyst for efficient CO_2_ reduction. Small.

[17] Xia W., Ren Y.Y., Liu J., Deng B.Y., Wang F. (2022). Non-synergistic photocatalysis of CO_2_-to-CO conversion by a binuclear complex of rigidly linking two cobalt catalytic centers. J. Photochem. Photobiol. A Chem..

[18] Jing H.W., Zhao L., Song G.Y., Li J.Y., Wang Z.Y., Han Y., Wang Z.X. (2023). Application of a mixed-ligand metal-organic framework in photocatalytic CO_2_ reduction, antibacterial activity and dye adsorption. Molecules.

[19] Xin X., Ma N., Hu C.Y., Liang Q., Bian Z.Y. (2019). Abundant manganese complex-anchored BiOI hybrid photocatalyst for visible light-driven CO_2_ reduction. NANO.

[20] Yasuomi Y., Takayuki O., Jun I., Shota F., Chinatsu T., Tomoya U., Taro T. (2019). Photocatalytic CO_2_ reduction using various heteroleptic diimine-diphosphine Cu(I) complexes as photosensitizers. Front. Chem..

[21] Fu Z.C., Mi C., Sun Y., Yang Z., Xu Q.Q., Fu W.F. (2019). An unexpected iron (II)-based homogeneous catalytic system for highly efficient CO_2_-to-CO conversion under visible-light irradiation. Molecules.

[22] Sakakibara N., Shizuno M., Kanazawa T., Kato K., Yamakata A., Nozawa S., Ito T., Terashima K., Maeda K., Tamaki Y. (2023). Surface-specific modification of graphitic carbon nitride by plasma for enhanced durability and selectivity of photocatalytic CO_2_ reduction with a supramolecular photocatalyst. ACS Appl. Mater. Interfaces.

[23] Tai X.S., Wang Y.F., Wang L.H., Yan X.H. (2023). Synthesis, structural characterization, hirschfeld surface analysis and photocatalytic CO_2_ reduction of Yb(III) complex with 4-aacetylphenoxyacetic acid and 1,10-phenanthroline ligands. Bull. Chem. React. Eng. Catal..

[24] Wang L.H., Tai X.S. (2023). Synthesis, structural characterization, hirschfeld surface analysis and photocatalytic CO_2_ reduction activity of a new dinuclear Gd(III) complex with 6-phenylpyridine-2-carboxylic acid and 1,10-phenanthroline ligands. Molecules.

[25] Liu W.J., Huang H.H., Ouyang T., Jiang L., Zhong D.C., Zhang W., Lu T.B. (2018). A copper(II) molecular catalyst for efficient and selective photochemical reduction of CO_2_ to CO in a water-containing system. Chem.-A Eur. J..

[26] Guo Z.G., Yu F., Yang Y., Leung C.F., Ng S.M., Ko C.C., Cometto C., Lau T.C., Robert M. (2017). Photocatalytic conversion of CO_2_ to CO by a copper(II) quaterpyridine complex. ChemSusChem.

[27] Liu S.Q., Zhou S.S., Chen Z.G., Liu C.B., Chen F. (2016). An artificial photosynthesis system based on CeO_2_ as light harvester and N-doped graphene Cu(II) complex as artificial metalloenzyme for CO_2_ reduction to methanol fuel. Catal. Commun..

[28] Liu P., Wang L.H., Tai X.S. (2023). The crystal structure of *catena*-poly[bis(6-phenylpyridine-2-carboxylato-κ^2^N,O)-(μ_2_-4,4′-bipyridne-κ^2^*N:N*)cadmium(II)], C_34_H_24_N_4_O_4_Cd. Z. Für Krist. New Cryst. Struct..

[29] Feng Y.M., Tai X.S., Xia Y.P. (2022). The crystal structure of [(2,2′-bipyridine-k^2^N,N)-bis(6-phenylpyridine-2-carboxylate-k^2^ N,O)copper(II)], C_34_H_24_N_4_O_4_Cu. Z. Für Krist. New Cryst. Struct..

[30] Tai X.S., Wang Y.F., Wang L.H., Yan X.H. (2023). Synthesis, structural characterization, and photocatalytic CO_2_ reduction activity of a new Gd(III) coordination polymer with 6-phenylpyridine-2-carboxylic acid and 4,4’-bipyridine ligands. Bull. Chem. React. Eng. Catal..

[31] Cao S.H., Li X.Z., Gao Y., Li F.H., Li K.X., Cao X.X., Dai Y.W., Mao L.R., Wang S.S., Tai X.S. (2020). A simultaneously GSH-depleted bimetallic Cu(II) complex for enhanced chemodynamic cancer therapy. Dalton Trans..

[32] Spackman P.R., Turner M.J., McKinnon J.J., Wolff S.K., Grimwood D.J., Jayatilaka D., Spackman M.A. (2021). CrystalExplorer: A program for Hirshfeld surface analysis, vis-ualization and quantitative analysis of molecular crystals. J. Appl. Crystallogr..

[33] Dolomanov O.V., Bourhis L.J., Gildea R.J., Howard J.A.K., Puschmann H. (2009). OLEX2: A complete structure solution, refinement and analysis program. J. Appl. Crystallogr..

[34] Sheldrick G.M. (2008). A short history of SHELX. Acta Crystallogr..

[35] Sheldrick G.M. (2015). Crystal structure refinement with SHELXL. Acta Crystallogr..

